# The pilot-scale preparation of the SA-hGM-CSF bi-functional fusion protein

**DOI:** 10.1080/21655979.2019.1608712

**Published:** 2019-04-24

**Authors:** Xiaoqing Li, Shirong Zhou, Yao Wang, Hui Lian, Anxin Zuo, Kaihua Zhou, Ling Tong, Zhujun Zhou, Jimin Gao

**Affiliations:** Zhejiang Provincial Key Lab for Technology & Application of Model Organisms,School of Laboratory Medicine and Life Sciences, Wenzhou Medical University, Wenzhou, China

**Keywords:** SA-hGM-CSF fusion protein, purification, refolding, pilot-scale, optimization

## Abstract

The granulocyte-macrophage colony-stimulating factor (GM-CSF) can be used to induce a powerful immune response. Based on the specific binding of biotin and streptavidin, SA-hGM-CSF was anchored on the surface of biotinylated tumor cells, which could enhance the anti-tumor effect of tumor cell vaccines in our previous reports, suggesting it would have potential clinical value. Preparation of the biologically active proteins in large-scale production is the basis of clinical application, however, only a small amount of biologically active protein was obtained according to previous studies. In this study, we researched the effects of various factors on the purification and simultaneous renaturation of SA-hGM-CSF fusion protein by single factor experiment and orthogonal experiment. Here, we developed a viable pilot-scale trial in the fermentation, purification, refolding and freeze-drying of SA-hGM-CSF proteins in order to efficiently obtain more biologically active proteins with high purity, which will lay the foundation for industrial production.

## Introduction

Granulocyte macrophage colony stimulating factor (GM-CSF) is a hematopoietic growth factor and immune protein which can be used in vaccines and clinical treatment [1]. It contains 127 amino acid proteins formed by the antiparallel arrangement of the two β-sheet and four α-helix bundles [2]. Since 1986, the Amgen first used it in the clinical trials and then it has been received FDA approval in the United State. GM-CSF was widely used to treat cancer because of its good curative effects with little side effects [3]. It could promote and regulate proliferation and differentiation of macrophages, neutrophils, and monocytes and enhance the resistant function of eosinophils and mature neutrophils for tumor [4–6]. Moreover, GM-CSF can inhibit tumor angiogenesis and metastasis, and improve the survival rate of surgical adjuvant therapy of highly recurrent malignant melanoma in clinical studies [7]. In addition, GM-CSF can also be used as a tumor vaccine adjuvant to effectively enhance the immune effect of tumor vaccines [3,8].

Streptavidin (SA) is a soluble homo-tetrameric protein isolated from Streptomyces without any glycosylation [9]. It can bind with biotin in an extremely high affinity with the Kd value of 10 −15 M, 10^3^ to 10^6^ folds higher than that for typical antigen-antibody interaction [10]. In the previous study, we have built the cell membrane surface modification technology that the protein could anchor in the tumor lesion by specificity binding with the biotinylated protein of the cytomembrane surface to achieve a sustaining and effective anti-tumor response. The research in vivo showed significant effects in the therapy of the superficial bladder and prostate cancers by using the SA-hGM-CSF fusion protein, highlighting the opportunity of clinical application [11,12]. However, refolding and simultaneous purification of SA-hGM-CSF from inclusion bodies is difficult with low yields of bioactive protein being produced. Therefore, it is urgent to explore and optimize the optimal conditions for the preparation of SA-hGM-CSF on a large scale.

It is known that purifying and recovering proteins from inclusion bodies is tedious [13], requiring isolation and dissolution of the inclusion bodies and refolding the protein to get a natural function [14]. Refolding protein belongs to downstream biological engineering technology which allows the protein to spontaneously recover from a denatured state to a bioactive, stable, low-energy structure by reducing or even completely eliminating the concentration of the protein denaturant [15]. The gradient and concentration of urea would affect the renaturation process in the previous study. At the same time, different physical parameters such as pH, ionic strength and temperature may have an enormous impact on the efficacy of purification simultaneous renaturation processes in vitro [16]. In addition, protein concentration has a significant influence on renaturation yield as one of the crucial elements for successful renaturation. On account of the increased hazard of protein aggregation at high concentrations, refolding is usually required at high dilutions [17]. Whereas, renaturation at low concentrations is usually uneconomical for large-scale protein production. Consequently, purification simultaneous renaturation requires a good grasp of the sample load and flow rate, which directly affect the protein concentration. Studies have shown that numerous chemical additives could reduce the misfolding of proteins during the process of purification and renaturation. L-Arginine is one of the most widely used additives for renaturation of proteins which often increases the refolding yield significantly in concentrations between 0.2 and 1M [18]. Furthermore, proteins with disulfide bonds present an additional challenge because the renaturation of the protein depends on the formation of the correct disulfide bond. What’s more, the disulfides between the erroneous residues results in misfolding or aggregation of proteins [19]. A mixture of reduced (RS) and oxidized (RSSR) forms of low molecular weight thiol reagents (GSH/GSSG) was added which generally provides the correct redox potential to promote the formation of the correct disulfide bond and the ratio of GSH to GSSG should correspond to a redox potential that is compatible with protein disulfide formation [20]. Therefore, there were seven factors needing to study in a pilot plant including the pH of refolding buffer, length of urea gradient, sample flow velocity, the sample load, the GSH/GSSG ratio of refolding buffer, urea concentrate of refolding buffer, and the amount of L-arginine in the refolding buffer.

However, there is no omnipotent method to facilitate the purification simultaneous renaturation of any given protein. Several methods of protein renaturation have been exploited [6,7], which are based on dilution, dialysis or solid phase fixation for the refolding process [21,22]. According to reports, based on the methods of dialysis and dilution, various additives can ameliorate the recovery of protein renaturation [21]. Despite this, there are still some hurdles. The optimal concentration of additives that influence renaturation and the interaction between them is difficult to determine, especially for different proteins, so determining the optimal purification simultaneous renaturation factors may be difficult and time-consuming. Therefore, it is necessary to explore and optimize the method for the purification simultaneous renaturation of SA-hGM-CSF to make it more effective and simplified. Moreover, the methods used to determine refolding yields are generally not well defined and/or untrustworthy. Studies have shown that pulse renaturation processes [23], size exclusion chromatography and adsorption chromatography can increase recovery [24]. Protein renaturation by ion exchange chromatography (IEC) was introduced by Creighton in 1986 [25]. The renaturation system was comprised of three buffers, in which a decreasing gradient of urea concentration was used to renature protein, and then the renatured protein was eluted with an increasing gradient of salt concentration. IEC has been applied successfully for the purification simultaneous renaturation of some recombinant proteins expressed by E.coli. The outstanding advantage of ion exchange chromatography is efficient that can be directly automated, facilitating large-scale protein production.

In this study, we have demonstrated experimental procedures for the production of SA-hGM-CSF protein on a pilot scale, which has been explored and optimized through fermentation, purification, and renaturation. Finally, we get the optimal conditions that are repeatable and ideal for process scale operations. The highly efficient and time-saving pilot-scale production of SA-hGM-CSF protein will facilitate the industrial production of drugs.

## Result

### The pilot-scale fermentation of the SA-hGM-CSF fusion protein

**Figure 1.**
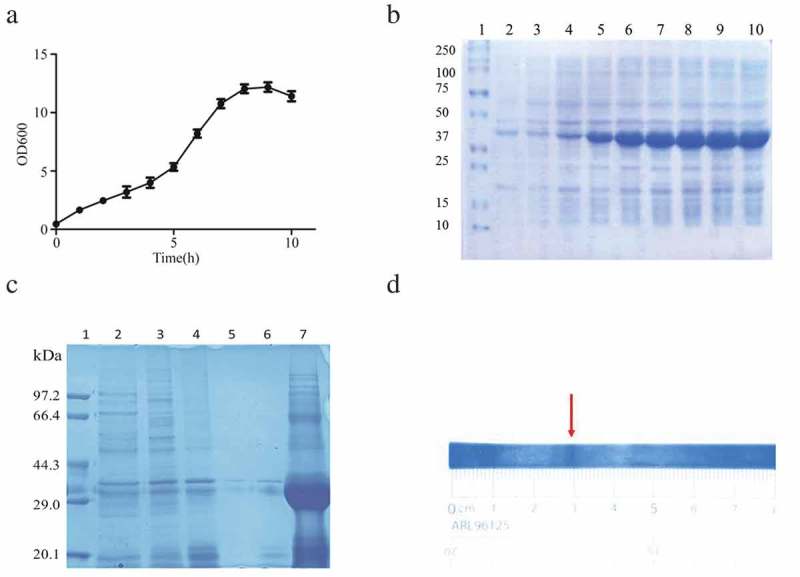
The production of SA-hGM-CSF. (a) The OD_600_ values of ferment medium and the relative expression of SA-hGM-CSF. (b) The optimization of induction time. 1: Marker; 2 ~ 10: The expression of SA-hGM-CSF after 1 ~ 9h. (c) The identification of inclusion bodies after washing. 1:marker; 2 ~ 6: The supernatant liquid of the inclusion body after washing with A,B,C,D,E buffer respectively; 7: The lysis buffer of the inclusion body. (d) The location of the SA-hGM-CSF protein in the isoelectric focusing electrophoresis.

The plasmid pET24a-SA-hGM-CSF was transformed into *E.coli* BL21(DE3), then it was cultured in a 10 L fermenter. When the OD_600_ reached about 3.0 (Figure 1(a)), IPTG was used to induce the expression of SA-hGM-CSF. The OD_600_ value reached peak after 10 h induction, the *E.coli* bacteria were harvested (Figure 1(b)). Finally, 30 g wet bacteria per liter medium was harvested and SA-hGM-CSF protein was expressed in the form of inclusion bodies, reaching 50% of the total bacterial population. Then the inclusion bodies were washed and dissolved in 8M urea (Figure 1(c)). Since the pH of the solution was important for protein solubilization, the theoretical pI of SA-hGM-CSF fusion protein was 5.87, calculated by ExPASy software. Simultaneously, the isoelectric point (pI) of the SA-hGM-CSF was detected by isoelectric focusing electrophoresis (IEF), the pI was 5.3032(Figure 1(d), S1A and S1B).

#### Effect of the single factor on purification and simultaneous refolding of the SA-hGM-CSF fusion protein

**Figure 2.**
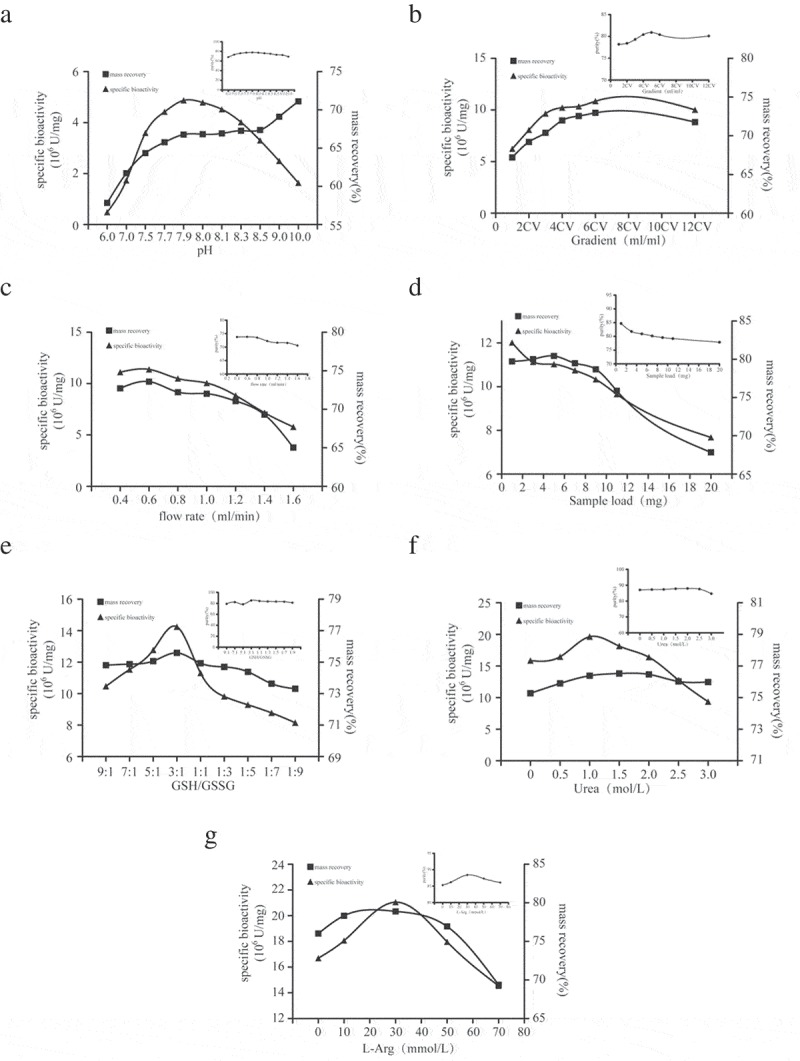
Single factor experiments. (a) The impact of different pH. (b) The impact of urea gradient. (c) The impact of flow rate. (d) The impact of sample load. (e) The impact of the GSH/GSSG. (f) The impact of urea concentration. (g) The impact of L-Arg concentration.

Based on the DEAE sepharose Fast Flow ion exchange chromatography, various factors affecting purification, biological activity, and recovery of renatured proteins need to be determined, including pH of refolding and elution buffer (Figure 2(a), Fig. S2A and B), urea gradient (Figure 2(b), Fig. S2C and D), sample flow velocity(Figure 2(c), Fig. S2E and F), the sample load of the SA-hGM-CSF (Figure 2(d), Figure S2G and H), the GSH/GSSG ratio of refolding buffer (Figure 2(e), Figure S2I and J), urea concentrate of refolding buffer (Figure 2(f), Fig. S2K and L), and the amount of L-arginine in the refolding buffer (Figure 2(g), Fig. S2M and N). According to the diagrams, when pH was 7.9, 4 column volume (CV) length of urea gradient, 0.6ml/min rate, GSH/GSSG = 3:1, 1M urea and 30mM L-Arg, would be beneficial for the purity, bioactivity and mass recovery of SA-hGM-CSF. However, sample load played a negative role.

### Pilot scale and test procedure for purification and simultaneous refolding of SA-hGM-CSF fusion proteins

**Figure 3.**
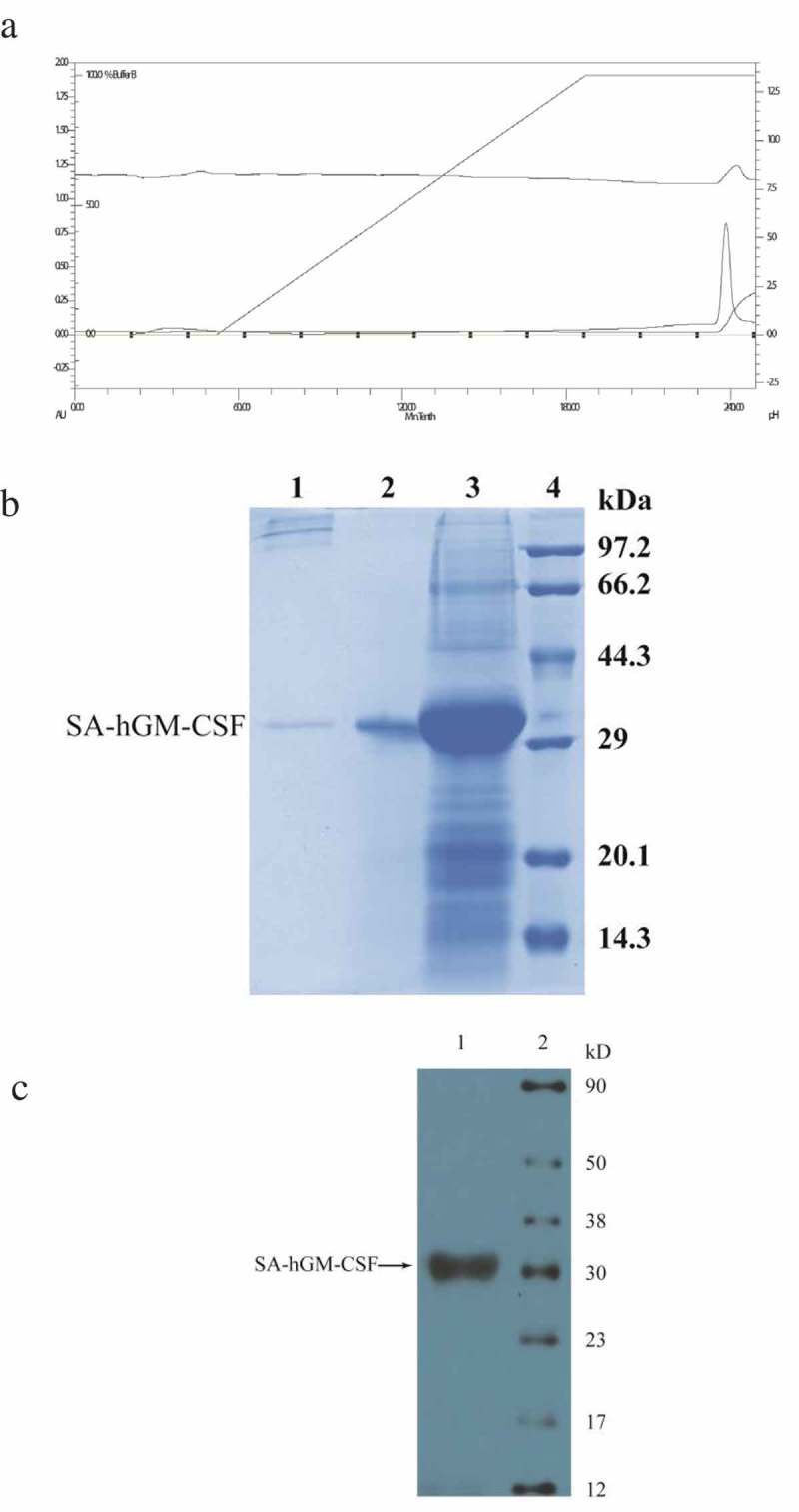
The purification and simultaneous refolding of the SA-hGM-CSF fusion protein. (a) The chromatogram of SA-hGM-CSF after purification and refolding . (b) The SDS-PAGE of SA-hGM-CSF after purification and refolding.1:After renaturation; 2: Reductive SA-hGM-CSF; 3:Before sample; 4:Marker. (c) The Western blot analysis of SA-hGM-CSF. Lane 1: SA-hGM-CSF. Lane: marker.

According to the single factor experiments, A 7-factor and 3-level orthogonal experiment were designed to optimize the conditions for SA-hGM-CSF purification and refolding. Finally, the optimum conditions were determined by the orthogonal test (Table 1), which was pH 7.9, 3.5cV urea gradient, 0.4ml/min, GSH/GSSG = 3:1, 1.5M urea and 30mM L-Arg, 0.7mg/ml sample load. On the basis of the small test, the XK30/50 column was used for the extended study of the pilot process. The solution was prepared under optimized conditions and the elution peak of SA-hGM-CSF protein was collected to determine protein concentration, purity and activity after equilibration, loading, re-equilibration, refolding and elution. The chromatogram was shown in Figure 3(a) and the results of SDS-PAGE were shown in Figure 3(b). Meanwhile, Western blot analysis showed that SA-hGM-CSF could bind to the monoclonal antibody and produce a color reaction, as shown in Figure 3(c). Moreover, the results of the three batches of pilot tests were listed in Table 2.

**Table 1.** Orthogonal design for purification and simultaneous renaturation of SA-hGM-CSF protein.

Column	1	2	3	4	5	6	7	8
Factor	pH	Gradient (ml/ml)	GSH/ GSSG	Urea (mol/L)	Flow rate (ml/min)	Sample load (mg/ml)	L-Arg (mM)	Bioactiv-ity(×10^6^ IU/mg)
Experiment 1	7.5	3.5	1:1	0.5	0.4	0.3	20	16.119
2	7.5	4	3:1	1	0.6	0.5	30	16.239
3	7.5	4.5	5:1	1.5	0.8	0.7	40	15.200
4	7.9	3.5	1:1	1	0.6	0.7	40	16.810
5	7.9	4	3:1	1.5	0.8	0.3	20	22.676
6	7.9	4.5	5:1	0.5	0.4	0.5	30	22.041
7	8.3	3.5	3:1	0.5	0.8	0.5	40	18.149
8	8.3	4	5:1	1	0.4	0.7	20	19.004
9	8.3	4.5	1:1	1.5	0.6	0.3	30	21.097
10	7.5	3.5	5:1	1.5	0.6	0.5	20	15.659
11	7.5	4	1:1	0.5	0.8	0.7	30	14.736
12	7.5	4.5	3:1	1	0.4	0.3	40	20.504
13	7.9	3.5	3:1	1.5	0.4	0.7	30	23.026
14	7.9	4	5:1	0.5	0.6	0.3	40	22.467
15	7.9	4.5	1:1	1	0.8	0.5	20	18.918
16	8.3	3.5	5:1	1	0.8	0.3	30	17.705
17	8.3	4	1:1	1.5	0.4	0.5	40	19.402
18	8.3	4.5	3:1	0.5	0.6	0.7	20	20.321
KF1/6	16.410	17.911	17.847	18.972	20.016	20.095	18.780	
KF2/6	20.989	19.087	20.152	18.197	18.765	18.401	19.140	
KF3/6	19.280	16.263	18.679	19.510	17.897	18.183	18.760	
R	4.5799	2.825	2.3055	1.3132	2.1187	1.9119	0.385

**Table 2.** The pilot-scale renaturation with simultaneous purification of the SA-hGM-CSF protein.

Times	One	Two	Three
Total protein before loading (mg)	358.6	336.2	348.6
Purity of target protein before loading (%)	65.7	63.2	64.5
Protein content after purification and renaturation(mg)	157.22	135.46	144.38
Purity of target protein after purification and renaturation (%)	95.7	96.8	95.2
The recovery rate of the target protein (%)	63.86	61.71	61.13

### Verification of the SA-hGM-CSF protein

**Figure 4.**
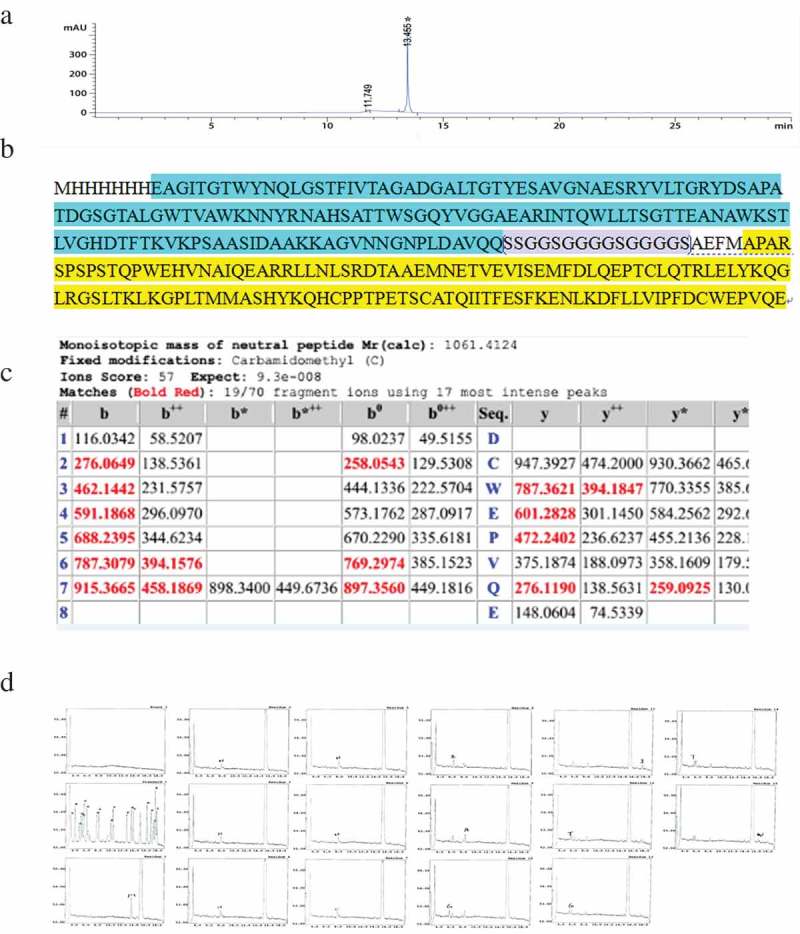
The characteristics of SA-hGM-CSF. (a) The purity analysis of SA-hGM-CSF protein by HPLC. (b) The theoretical sequence of SA-hGM-CSF protein. (c) The C-terminal domain of SA-hGM-CSF protein sequenced by MS. (d) The N-terminal domain of SA-hGM-CSF protein sequenced by MS.

After purification and renaturation under optimal conditions, some characteristics of SA-hGM-CSF need further verification. HPLC (Figure 4(a)) was used to detect the purity, which was above 95%. The C- and N-terminal sequences were determined by mass spectrometry (MS) and they were consistent with theoretical sequence (Figure 4(b-d)). The C-terminal sequence was DCWEPVQE and the N-terminal sequence was MHHHHHHEAGITGTW (Figure 4(c,d)).

### The bioactivity identification of the SA-hGM-CSF protein

**Figure 5.**
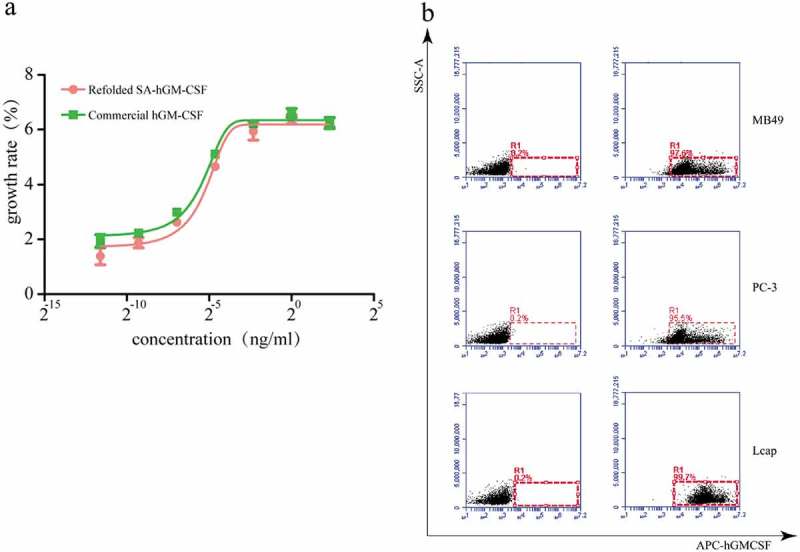
The bioactivity of SA-hGM-CSF protein (a) Proliferation rate of TF-1 cells. (b) The anchoring rate of SA-hGM-CSF protein was analyzed by flow cytometry, control (left): no SA-hGM-CSF.

As we all know, the proliferation of TF-1 cells depends on SA-hGM-CSF. Based on this, the biological activity of SA-hGM-CSF was identified by CCK-8 kit which was 5.6 × 10^7^ U/mg(Figure 5(a)). Meanwhile, the SA-hGM-CSF protein could efficiently and specifically bind with biotin by SA-domain. Biotinylated PC-3, MB-49, and LN cap cells were used to detect the anchoring rate of the fusion protein, which exceeded 98% (Figure 5(b)).

### Freeze-drying and preservation

**Figure 6.**
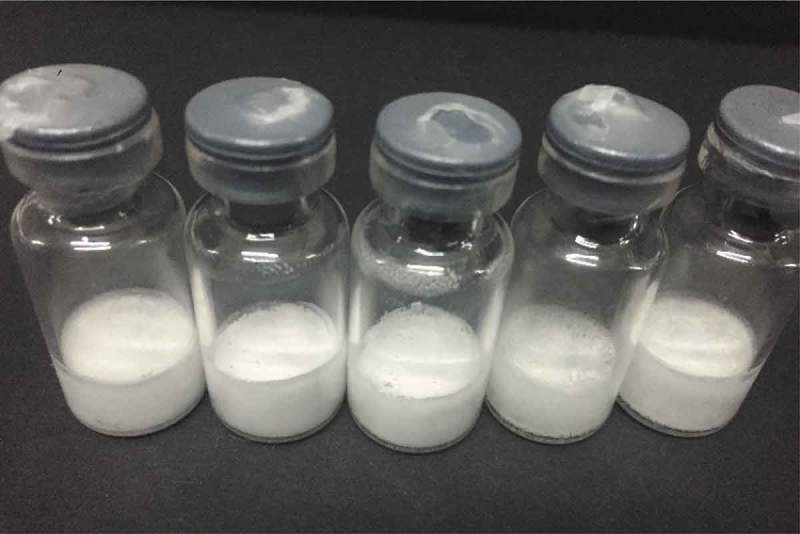
The freeze-drying of SA-hGM-CSF protein after purification and refolding.

For convenient transportation and long-term storage, we freeze-dried the SA-hGM-CSF protein through a lyophilizer (Figure 6). After a period of time, the freeze-dried protein was stored at 4°C or room temperature. Then we re-dissolved the protein in PBS buffer, identified its biological activity and purity according to previous experiments, and found no significant changes.

## Discussion

The studies in this paper focused on exploring and optimizing the conditions for the large-scale production of biologically active proteins. In this work, an anion exchange chromatography method was used to explore the single factor affecting the purification and refolding of SA-hGM-CSF protein and optimized by orthogonal experiment. With the determined optimal conditions, the pilot process for the purification and simultaneous refolding of SA-hGM-CSF protein was carried out successfully to obtain high purity (more than 95%) with a high recovery rate more than 60%, and endotoxin content less than 10EU/unit. Our results suggested the dual function fusion protein was obtained effectively in a short time by the method of purification and simultaneous column renaturation, which laid a solid foundation for the subsequent large-scale production and clinical research.

Compared with the traditional refolding methods such as dilution method and dialysis method, ion exchange chromatography shows unique advantages in protein purification and refolding. The dilution method can’t remove impurity protein and the follow-up process is complex though it is convenient. Although the dialysis renaturation method can obtain more stable proteins, it can only remove small amounts of small-molecule impurity proteins. At the same time, it takes longer and is limited to the volume of the dialysis bag, which cannot extend its application [22]. Ion exchange chromatography has a good application prospect for the large scale amplification of purified and renatured proteins because it controls the process and is not limited by equipment, after which we can concentrate proteins and remove endotoxins. In addition, this method of purification and simultaneous column refolding saves time and can efficiently obtain the desired protein.

However, we should avoid using guanidine hydrochloride solution to dissolve the protein, because guanidine hydrochloride can increase the ionic strength of the protein that is suspended on the column, and it can also increase the ionic strength of L-Arg. In addition, a good redox environment and reduction of the aggregate formation for the refolding of protein are pivotal to improve the activity of the protein.

Here, we introduced the redox couple (GSH/GSSG) in the refolding buffer to provide a better environment for protein renaturation and joined the arginine to reduce the formation of protein aggregates. The mechanism of arginine about how to affect the refolding process of the protein has not yet been elucidated, maybe because it decreases the aggregative formation of protein by the reaction with protein tryptophan side chain. Moreover, it is necessary to use high-quality raw materials, whose low impurity and high purity features which will bring better results.

According to previous studies, it is reported that human drugs would be better without tissue markers, so a histidine-free SA-hGM-CSF protein strain is proceeding to be constructed in our laboratory. The SA-hGM-CSF protein has been used for the prevention and treatment of mouse bladder and prostate cancer models, which showed good efficacy in our previous studies. It is well known that hGM-CSF can not only treat cancer but also treat other diseases. In addition, hGM-CSF can also heal wounds, diagnose, prevent and treat infections caused by bacteria, viruses, fungi, and parasites [26,27]. Furthermore, it also plays an important role in the physical recovery of patients undergoing autologous bone marrow transplantation [28]. Similarly, our improved protein purification and the refolding process will also facilitate the treatment of these diseases.

To summarize, we have elucidated pilot-scale trials in the fermentation, purification, refolding, and freeze-drying of SA-hGM-CSF proteins. In addition, we successfully optimized protein purification and renaturation conditions and prepared SA-hGM-CSF with dual functions and broad application prospects, which will contribute to the production of prostate cancer vaccines and bladder cancer vaccines.

## Materials and methods

### Cell lines, plasmids and reagents

Plasmid PET24a(+)-SA-hGM-CSF was constructed and maintained in our laboratory. The human erythroleukemia cells (TF-1) and prostate cancer cell line LNCaP as well as PC-3 were purchased from the cell bank of Chinese Academy of Sciences (Beijing, China), and maintained in DMEM/F12 (HyClone，USA). containing 10% (v/v) fetal bovine serum (Gibco，USA), 1% penicillin/streptomycin(Gibco). The mouse bladder cancer cell line (MB49) was purchased from American Type Culture Collection (ATCC), and cultured in RPMI1640 (Gibco) supplemented with 10% fetal bovine serum (Gibco), 1% penicillin/streptomycin(Gibco), at 37°C, in 5% CO_2_ humidified incubator. Tris (hydroxymethyl) aminomethane, Sodium chloride, Sodium dodecyl sulfate, Ethylenediaminetetraacetic acid, Urea, Glycine, and L-Arginine were obtained from Sangon Biotech Shanghai Co Ltd. The other reagents used in this study were of analytical grade and are commercially available.

### Expression of the SA-hGM-CSF protein in e.coil

The plasmid PET24a(+)-SA-hGM-CSF was transformed into the BL21(DE3) Competent cells. 5μL BL21(DE3)-PET24a-SA-hGM-CSF bacterial liquid was injected into the 10 ml LB liquid medium containing 50 μg/mL kanamycin (Beyotime), then the LB liquid medium was incubated overnight at 37°C with shaking on the shaker (IKA KS 4000 I control, Germany) and next inoculated into 500 mL culture medium containing kanamycin in the following night. It was inoculated into a 10L fermentor (NBS, BIOFLO 415, USA) and fermented in a ratio of 1:20 at OD_600_ = 1.5 . Through the previous exploration of the induction conditions, we found that it is best to add IPTG (Beyotime) to a final concentration of 0.5 mM at OD_600_ = 3 and continued for 10 hours. Finally, the cells were harvested by centrifugation at 4000 rpm at 4°C for 15 minutes (Beckman Coulter, Avanti j-26 XP, USA).

### Isolation of the inclusion bodies

\Wet cell paste was resuspended at a ratio of 1/20 (w/v) in bacterial lysis buffer (50 mmol/L Tris-HCl, 1 mmol/L EDTA, 100 mmol/L NaCl, and 1 mg/ml lysozyme, pH8.0) and crashed by cell crusher (JNBIO,China) four passages through the press at 1000–1500 MPa in an ice-water bath and then the pellet containing inclusion bodies (IBs) was collected by centrifugation at 12,000 rpm at 4°C for 30 min(Thermo Science, USA). Then, the isolated inclusion body pellet was dissolved at a ratio of 1/20 (w/v) in washing buffer A (20mM Tris-HCl, 2mM EDTA,0.5M NaCl) and stirred washing on the magnetic stirrer (Kylin-Bell.GL-3250C, China) for 3 h at 4°C. After 3 h stirring, the inclusion bodies were harvested by centrifugation at 12000 rpm at 4°C for 30 minutes(Thermo Science), next, solubilized in resuspension buffer B (20 mM Tris-HCl, 10mM EDTA, 2mM β-ME, 0.10% Triton X-100), buffer C (20mM Tris-HCl, 10mM EDTA, 2M urea), buffer D (20mM Tris-HCl, 2mM EDTA, 50% Isopropanol) and buffer E (20mM Tris-HCl) to repeat the step to wash the inclusion body, afterward, dissolved in the 8M urea buffer (30mM Tris-HCl, 1mM EDTA, 8M urea, 5mM β-ME) with pH 8.0 and stirred on the magnetic stirrer at 4°C for overnight. Ultimately, the soluble fraction containing the denatured protein of interest was obtained by centrifuging at 12000rpm at 4°C for 30 minutes(Thermo Science) and stored at −20°C. The supernatant of all the above steps was tested with SDS-PAGE.

### Purifying and refolding the SA-hGM-CSF protein by ion exchange chromatography(IEC)

The final dissolved protein supernatant solution was filtered on a 0.22 µm filter (Millipore, USA) and loaded onto a 10ml DEAE Sepharose Fast Flow ion-exchange column (GE Healthcare Life Sciences, USA) which was connected to AKTA Purifier 100 (GE, USA) as well as initially equilibrated with buffer solution A. The column was rebalanced and ran from 100% balance fluid to 100% refolding buffer (solution B) to form linear gradient to induce renaturation, and then continued to run 2CV (column volume) refolding buffer. Finally, the refolded protein was eluted by elution buffer (solution C, refolding buffer + 1M NaCl), then the elution peak containing the protein of interest was collected.

Mobile phase I: Buffer A: 30 mmol Tris-HCl + 1 mmol EDTA. Buffer B: 30 mmol Tris-HCl + 1 mmol EDTA. Buffer C: 30 mmol Tris-HCl + 1 mmol EDTA + 1 mol NaCl. Buffer solutions A, B, and C were adjusted to different pH values.

Mobile phase II, III, IV, and V: Buffer A: 30 mmol Tris-HCl + 1 mmol EDTA(pH 7.9). Buffer B: 30 mmol Tris-HCl + 1 mmol EDTA (pH 7.9). Buffer C: 30 mmol Tris-HCl + 1 mmol EDTA + 1 mol NaCl (pH 7.9). We investigated the effects of urea gradient length, flow rate and sample loading on protein purification and refolding, respectively.Subsequently, the ratio of GSH/GSSG in buffer B and C was 9:1, 7:1, 5:1, 3:1, 1:1, 1:3, 1:5, 1:7, 1:9, respectively.

Mobile phase VI: Buffer A: 30 mmol Tris-HCl + 1 mmol EDTA (pH 7.9). Buffer B: 30 mmol Tris-HCl + 1 mmol EDTA+ 3mmol GSH + 1mmol GSSG (pH 7.9). Buffer C: 30 mmol Tris-HCl + 1 mmol EDTA +3mmol GSH + 1mmol GSSG + 1 mol NaCl (pH 7.9) .The final concentration of urea was 0,0.5, 1,1.5, 2,2.5, 3M in buffer B and C, respectively.

Mobile phase VII: Buffer A: 30 mmol Tris-HCl + 1 mmol EDTA (pH 7.9). Buffer B: 30 mmol Tris-HCl + 1 mmol EDTA+ 3mmol GSH + 1mmol GSSG +1 mol urea (pH 7.9). Buffer C: 30 mmol Tris-HCl + 1 mmol EDTA +3mmol GSH + 1mmol GSSG+1 mol urea + 1 mol NaCl (pH 7.9) .The concentration of L-arginine was 0, 10, 30, 50, 70, 100 mM in buffer B and C, respectively.

### HPLC and mass spectrometry

The purified and refolded protein was digested individually with protease, and peptides were separated and analyzed by LC-MS/MS. The purity of the protein was analyzed by an Agilent 1260 Infinity HPLC system (Agilent Technologies, Palo Alto, CA, USA) equipped with a manual injector and diode array detector (DAD). An Aeris WIDEPORE C18 column (150 mm × 4.6 mm, 5 µm particle size) was used, and all injections were performed manually through a 20 µL sample loop. The mobile phase consisted of acetonitrile-trifluoroacetic acid (99.9/0.1, v/v) and water-trifluoroacetic acid (99.9/0.1, v/v), which were designated as A and B, respectively. The gradient elution program followed: 4 min, 25%A; 10 min, 55%A;16 min, 75%A and 18 min 100%A. The flow rate was 0.8–1 mL/min, the column temperature was kept at 25°C, and the detector wavelength was set at 280 nm. Chromatographic data were acquired and processed using Xcalibur software. The purity was calculated by analyzing the ratio of peak area.

### SDS-PAGE and western blotting

SDS-PAGE was used to evaluate the purity of the protein. The reduced samples need to be boiled. Comparing the reducing and non-reducing conditions, the renaturation efficiency can be easily verified for proteins containing disulfide bonds, Proteins were analyzed by 12% gel and stained with Coomassie Brilliant Blue (Sangon).

Western blotting was used to identify the specificity of proteins. After electrophoresis, the protein was transferred to the nitrocellulose membrane for Western blot analysis. The membrane was sealed with 5% skim milk (BD, USA) and incubated at room temperature for 1 hour, washed three times with TBST, then incubated the mouse anti-human GM-CSF monoclonal antibody (R&D systems, Minneapolis, MN) overnight at 4 °C, next, washed three times, and incubated goat anti-mouse IgG (Proteintech, USA) conjugated with horseradish peroxidase at 37 °C for 1 hour. Afterward, the HRP activity was demonstrated by enhanced chemiluminescence (Millipore, USA).

The glue and membrane were scanned on a BioRad GelDoc XR+ System (USA) and band intensities were analyzed by the ImageLab software.

### Bioactivity assay

The bioactivity of SA-hGM-CSF fusion protein was evaluated by TF-1 cell proliferation assay and flow cytometry. In accordance with the method described in previous studies, the biological activity of rhGM-CSF was determined by the CCK8 method based on the cytokine-dependent cell line TF-1. The cells were first starved for 3 days and inoculated in 96-well plates (Corning, flat and clear bottom)at a density of 10^5^cells/ml. then SA-hGM-CSF was diluted in multiples and added to the corresponding wells, and each hole has three duplicate holes. RhGM-CSF (PeproTech, USA) was the positive control group. Simultaneously, no cytokines were added to the negative control group, and the only medium was added to the blank control group. The cells were cultured for 3 days in a 37 °C, 5% CO _2_ incubator, and 20 µl CCK8 (Dojindo Lab, Japan) was added 4 h before the end of incubation. Finally, the absorbance was read at 450 nm using an Absorbance Microplate Reader (ThermoForma, USA). Non-linear regression curve fit the dose-response curves were analyzed by the Prism 6 (GraphPad) to determine the EC_50_ values.

To examine the biotin-binding activity of SA-hGM-CSF fusion protein, PC-3, MB-49 and LNcap cells (2 × 10^5^) were incubated in freshly-prepared EZ-Link Sulfo-NHS-LC-LC-Biotin (Pierce, Rockford, IL, USA) at a final concentration of 0.05 µg/µl in PBS for 30 min in the 37 °C, 5% CO_2_ incubator, respectively. The cells were then washed with 1 × PBS containing 100mM glycine to remove the excess biotin and incubated with SA-hGM-CSF fusion protein (10 µg) in PBS for 1 h at room temperature, next, washed three times and stained with rat anti-human GM-CSF monoclonal antibody (BioLegend, San Diego, USA), the control was protein-free. What calls for special attention is that the procedure should be protected from light. At last, the cells were washed and filtered by the nylon membrane, and the anchor rate of the cells was acquired using the flow cytometer (BD FACSAriaTM III).

### Detection of the bacterial endotoxin

The limulus reagent was used to detect the bacterial endotoxin of the SA-hGM-CSF protein. Endotoxin standard protein solution and bacterial endotoxin positive control solution, as well as the sample solution, were prepared respectively. The operation procedure of the test was carried out strictly according to the instruction.

### Freeze-drying and preservation

The concentration of the purified and refolding SA-hGM-CSF protein was adjusted to 1 mg/ml, meanwhile, 5% mannitol (Sangon) and 1% trehalose protecting agent was added and mixed. The protein was subpackage (1 ml/vial), placed in the −80 °C refrigerator (Thermo, USA) for 24 h, then placed in a lyophilizer Four-Ring Science Instrument Plant Beijing CO., LTD, LGJ-18, China) with a set procedure for lyophilization overnight. Afterward, the bottle was sealed and stored at 4 °C.

### Statistical analysis

All values were expressed as mean ± s.d.unless otherwise indicated. All data statistics were used unpaired T-tests, all experiments were repeated at least three times. One of them was orthogonal experimental analysis. Polyline images were analyzed using GraphPad Prism 6 statistical software, and flow cytometry results were analyzed using FlowJo software.

## Highlights

1. The SA-hGM-CSF fusion protein was obtained by pilot-scale fermentation.
2. A 7-factor and 3-level orthogonal experiment showed optimal conditions for purification and refolding.
3. SA-hGM-CSF in pilot scale with optimized conditions had good bioactivity and high purity.
4. The method for preparation of SA-hGM-CSF was time-saving and more efficient.
